# Perspectives on long-acting growth hormone therapy in children and adults

**DOI:** 10.20945/2359-3997000000190

**Published:** 2019-11-01

**Authors:** Rayhan A. Lal, Andrew R. Hoffman

**Affiliations:** 1 Division of Endocrinology Department of Pediatrics Stanford University School of Medicine Stanford California USA Division of Endocrinology, Department of Pediatrics, Stanford University School of Medicine, Stanford, California, USA; 2 Division of Endocrinology Department of Medicine Stanford University School of Medicine Stanford California USA Division of Endocrinology, Department of Medicine, Stanford University School of Medicine, Stanford, California, USA; 3 Department of Veterans Affairs VA Palo Alto Health Care System Palo Alto California USA Medical Service, Department of Veterans Affairs, VA Palo Alto Health Care System, Palo Alto, California, USA

**Keywords:** Growth hormone, recombinant human growth hormone, pharmacology, review, drug development

## Abstract

Growth hormone therapy with daily injections of recombinant human growth hormone has been available since 1985, and is shown to be safe and effective treatment for short stature in children and for adult growth hormone deficiency. In an effort to produce a product that would improve patient adherence, there has been a strong effort from industry to create a long acting form of growth hormone to ease the burden of use. Technologies used to increase half-life include depot formulations, PEGylated formulations, pro-drug formulations, non-covalent albumin binding growth hormone and growth hormone fusion proteins. At present, two long acting formulations are on the market in China and South Korea, and several more promising agents are under clinical investigation at various stages of development throughout the world. Arch Endocrinol Metab. 2019;63(6):601-7

## INTRODUCTION

The era of growth hormone replacement began in 1958 when Raben used human growth hormone purified from pituitary glands that had been obtained at autopsy ( 1 ). Subsequently, the United States National Pituitary Agency and other national groups were organized to supervise the collection of human pituitary glands and the purification of human growth hormone from these glands. Since it was a scarce and precious resource, relatively few patients could be treated with these impure preparations ( 2 ), necessitating less than optimal dosing schedules. Moreover, supplies were occasionally interrupted. When it was discovered that a young man treated at Stanford with cadaveric human growth hormone since the age of 3 died of Creutzfeld-Jakob disease, the use of cadaveric growth hormone was terminated in most of world in 1985 ( 3 ).

Recombinant human growth hormone was made available later that year for the treatment of children. For the first time, there was an unlimited supply of growth hormone, and daily administration became the standard treatment. Soon, its use was not limited to severe growth hormone deficiency, and ultimately the drug was approved for milder forms of growth hormone deficiency, Turner syndrome, children born short for gestational age, and other causes of short stature including idiopathic short stature. Moreover, the unlimited supply led investigators to explore the use in adults with hypopituitarism. Adult growth deficiency syndrome was recognized and treated with recombinant human growth hormone therapy ( 4 , 5 ).

Adherence to a daily subcutaneous injection of any medication may become problematic, and several studies have shown that most children prescribed recombinant human growth hormone miss a substantial number of injections ( 6 , 7 ). Not surprisingly, those who were least adherent to the medication had less satisfactory growth. Many adults with adult growth hormone deficiency balk at giving themselves a daily shot, finding it inconvenient to travel with a syringe and a refrigerated drug ( 8 ). Therefore, it was postulated that a longer-acting forms of growth hormone that could be administered less frequently could result in better adherence to therapy and would be a more attractive mode of therapy to those who were unwilling to consider daily injections ( 9 ).

However, there were theoretical concerns that a long acting growth hormone preparation would be ineffective as a replacement therapy or would lead to over-treatment and the development of acromegaly. Growth hormone has a half-life of 10-20 minutes, is secreted in pulses throughout the day, with an increased number of pulses during sleep. Worries that constant, non-pulsatile exposure to growth hormone would lead to downregulation or desensitization of growth hormone receptors were allayed by studies in which subjects who received continuous subcutaneous infusions of growth hormone for up to 6 months were able to maintain normal serum IGF-I levels and to avoid any signs or symptoms of acromegaly ( 10 ). As a result, many companies began to explore novel strategies to develop long-acting analogs for use in both children and adults. We will discuss a representative group of these novel growth hormone formulations that have been tested in humans.

## DEPOT FORMULATIONS

Genentech (South San Francisco, USA) marketed the first recombinant human growth hormone product and was the first to gain approval for a long-acting growth hormone in 1999. Nutropin Depot, a preparation composed of native recombinant human growth hormone encapsulated in biodegradable poly (lactide coglycolide) copolymer microspheres, was manufactured in collaboration with Alkermes (Dublin, Ireland). This formulation was very viscous, and it required a large bore needle. Children require a relatively high dose of growth hormone (compared to adults) to achieve optimal growth, it often required more than a single injection at each dosing interval. Each injection left a visible subcutaneous lump. Monthly or twice weekly injections were adequate to produce annualized growth rates of 8.4 cm/year after 6 months in prepubertal children ( 11 ). The drug was also just as effective as daily recombinant human growth hormone in reducing visceral and truncal adipose tissue in adults with growth hormone deficiency ( 4 ), but it was withdrawn from the market in 2004.

Somatropin Biopartners (LB03002 or Declage) was formulated by BioPartners (Los Angeles, USA) and LG Life Sciences (Seoul, South Korea). The product comes in a powder containing recombinant growth hormone in sodium hyaluronate microspheres that are reconstituted in medium-chain triglycerides (MCT) oil. Tissue hyaluronidase breaks down the microspheres, slowly releasing growth hormone. In 2006, the pharmacokinetics and pharmacodynamics were evaluated in 6 men and 3 women being treated on a stable regimen for growth hormone deficiency. Participants underwent a 4-week washout followed by weekly administration of Somatropin Biopartners for five weeks. As compared to daily therapy, the depot formulation exhibited double the maximum serum growth hormone concentration, comparable dose-normalized GH area under curve, 34-41% increase in maximal serum IGF-1 and similar dose-normalized IGF-1 area under curve ( 12 ). Peter and cols. performed a randomized, controlled, investigator masked phase 2 study in 2009 on 37 pre-pubertal, children not previously on growth hormone. For 7 days, they received daily growth hormone 0.03 mg/kg, followed by a 3-week washout and then different weekly doses of Somatropin Biopartners for 3 months. As compared to daily therapy, the depot formulation exhibited 4-fold maximal serum growth hormone concentration with stable area under curve over 3 months. Normal IGF-1 standard deviation scores were achieved after 3 months and normal IGFBP-3 standard deviation scores after 1 week therapy ( 13 ). Biller and cols. performed a 26 week placebo-controlled trial of Somatropin Biopartners among 152 adults with growth hormone deficiency, there was a substantial increase in IGF-1 with reduced fat mass on DXA ( 14 ) that was sustained in a 1-year follow-up study ( 15 ). A substudy of the phase III trial showed significant reduction in fat mass and leptin with increased ghrelin without change in glucose or lipid metabolism ( 16 ). In 2012, a 3-year trial was published examining the efficacy and safety of Somatropin Biopartners 0.5-0.7 mg/kg/wk versus daily growth hormone in previously untreated prepubertal children with growth hormone deficiency ( 17 ). A 1-year pediatric trial in Korea demonstrated comparable mean height velocity, subsequently replicated in the 2-year phase III multinational trial ( 18 , 19 ). In 2013, LB03002 was approved in Europe, but not marketed for 3 years and so authorization lapsed. It is available for use in children in South Korea. A subsequent Korean multicenter, randomized, open-label phase II study in previously untreated prepubertal children with idiopathic short stature established 0.7 mg/kg/week as non-inferior to daily growth hormone 0.05 mg/kg ( 20 ).

## PEGYLATED FORMULATIONS

Polyethylene glycol (PEG) is a biologically inert and non-immunogenic compound that can be added moieties to proteins to extend their half-life in the circulation. PEGylated proteins like pegvisomant, a growth hormone antagonist, is a mainstay in the treatment of acromegaly. Numerous other PEGylated proteins have been developed to extend their half-lives in order to treat a host of diseases, including anemia and hepatitis. Clinical studies indicated that ARX201, a pegylated recombinant human growth hormone, made by Ambrx (La Jolla, USA), could raise and maintain serum IGF-I levels in adult growth hormone deficiency ( 21 ). However, drug development ceased when it was discovered PEG had accumulated in the ependymal cells of the choroid plexus in preclinical primate studies. Other companies, which were studying their own pegylated growth hormone preparations, terminated those programs.

In January 2014, GeneScience Pharmaceuticals’ (Changchun, China) PEGylated weekly growth hormone called Jintrolong, was approved in China for pediatric growth hormone deficiency. In a phase 1 trial, 12 children with growth hormone deficiency received daily growth hormone 0.0286 mg/kg for 7 days, followed by 4 weeks washout and weekly Jintrolong 0.2 mg/kg for 6 weeks. Jintrolong exhibited comparable serum IGF-1 and slower clearance than daily growth hormone without significant accumulation ( 22 ). Phase 2 and 3 trials were performed in 6 hospitals in China among previously untreated children with growth hormone deficiency. The phase 2 trial enrolled 108 participants and established safety and efficacy of Jintrolong at 0.2 mg/kg weekly for 25 weeks versus 0.1 mg/kg weekly dosing and daily growth hormone. The phase 3 trial enrolled 343 participants for 25 weeks and demonstrated greater height velocity increase and standard deviation height scores for weekly Jintrolong versus daily growth hormone ( 23 ). Two single-center, randomized open-label studies out of Guanzhou, China assessed tolerability of Jintrolong among 34 health adult subjects. Doses up to 0.8mg/kg were well tolerated. Pharmacokinetic profiles and plasma growth hormone concentrations supported potential for weekly administration ( 24 ).

## PRO-DRUG FORMULATIONS

Ascendis Pharma (Hellerup, Denmark) TransCon sustained-release growth hormone consists of growth hormone bound via proprietary linker to an inert carrier, methoxypolyethylene glycol (mPEG). The linker undergoes controlled pH and temperature dependent breakdown, releasing active growth hormone; it is excreted renally. A phase 1 randomized trial among 44 healthy male subjects compared 4 different doses of weekly TransCon Growth Hormone to 2 different doses of daily growth hormone. TransCon Growith Hormone was well tolerated with no binding antibody formation and comparable measures of serum growth hormone and IGF-1 ( 25 ). Phase 2 trials among 37 adults with growth hormone deficiency and 53 previously untreated prepubertal children with growth hormone deficiency revealed similar safety and efficacy of weekly TransCon Growth Hormone and daily growth hormone ( 26 , 27 ). The results of the Phase 3 heiGHt trial (NCT02781727) were presented at ENDO 2019 in New Orleans ( 28 ). 161 previously untreated prepubertal children with growth hormone deficiency received either weekly TransCon Growth Hormone 0.24 mg/kg or daily Genotropin 0.034 mg/kg. Annualized height velocity after 1 year was greater (p = 0.0088) in the TransCon Growth Hormone group (11.2 cm) versus Genotropin (10.3 cm). The Phase 3 fliGHt trial (NCT03305016) is evaluating use in younger participants and those who have used daily growth hormone in the past. Ascendis reports that based on this data and from the ongoing fliGHt and enliGHten (long-term extension) trials they expect to file a Biologics License Application in the first half of 2020.

## NON-COVALENT ALBUMIN BINDING GROWTH HORMONE

Novo Nordisk (Bagsvaerd, Denmark) manufactures Somapacitan (previously NNC0195-0092), growth hormone with a single point mutation to which a noncovalent albumin-binding terminal fatty acid is linked. Half-life is predicted to increase through albumin binding and subsequent reduced clearance ( 29 ). The same technology is used in the long-acting insulin detemir. A randomized, placebo-controlled trial of 105 healthy male subjects was conducted at Profil in Germany. The drug was well tolerated and demonstrated a dose-dependent increase in serum IGF-1 ( 30 ). A phase 1 trial among 34 growth hormone deficient men demonstrated tolerability and comparable serum IGF-1 to daily growth hormone ( 31 ). Similar findings were reported in the phase 1 trial of 32 previously treated growth hormone deficient prepubertal children ( 32 ). A phase 3, 26-week randomized, controlled trial performed in 6 countries recruited 92 adults with growth hormone deficiency. They were treated with weekly Somapacitan or daily Norditropin administered subcutaneously by pen. Somapacitan was well-tolerated, IGF-1 standard deviation scores remained therapeutic, and participants felt dosing was more convenient ( 33 ). Pediatric phase 3 testing (REAL4 trial – NCT03811535) began in 2019 and is expected to conclude in 2024.

## GROWTH HORMONE FUSION PROTEINS

Genexine (Seongnam, South Korea) and Handok (Seoul, South Korea) manufactures HyTropin (GX-H 9 ), a chimeric protein of growth hormone fused to the Fc-domain of immunoglobulin. This hybrid Fc (hyFc) is created from IgD, which provides flexibility and low antibody-dependent cellular cytotoxicity, along with IgG4 which binds to the protective neonatal Fc receptor and exhibits low complement-dependent cytotoxicity ( 34 ). HyTropin’s expanded size reduces renal clearance and increases half-life. A Phase 2 randomized, open-label study was conducted in 16 endocrinology centers in Europe and Korea among 45 adult participants with growth hormone deficiency. With twice-monthly administration, the drug demonstrated comparable efficacy and acceptable tolerability without significant immunogenicity ( 35 ). The manufacturer is planning a Phase 3 study with a longer treatment period and more patients in the United States.

OPKO Health (Miami, USA) and Pfizer (New York City, USA) manufacture Somatrogon, Lagova or MOD-4023, a chimeric product generated by fusing three copies of the 28 carboxy-terminal residues of human chorionic gonadotropin (hCG) beta subunit to the coding sequence of growth hormone ( 36 ). The Phase 2 study of weekly MOD-4023 in 54 adults with growth hormone deficiency revealed safety and comparable serum IGF-1 values to daily growth hormone ( 37 ). A Phase 2 study among 53 prepubertal children with growth hormone deficiency receiving weekly MOD-4023 for one year demonstrated safety and efficacy with 6 month annualized height velocity above 12 cm/year ( 38 ). Subsequent pharmacokinetic and pharmacodynamic studies in children established that sampling 4 days following dose administration allowed best estimation of mean IGF-1 SDS ( 39 ). In December 2016 OPKO reported phase 3 trial results in growth hormone deficient adults. The initial findings demonstrated no statistical difference between MOD-4023 and placebo treated subjects in the primary endpoint of trunk fat mass from baseline to 26 weeks. Post-hoc sensitivity analyses excluding outliers did show a statistically significant difference. Phase 3 trials for pediatric growth hormone deficiency are ongoing.

TV-1106, an analog that fused recombinant human growth hormone to albumin ( 40 ), was initially tested in growth hormone deficient children but was withdrawn by Teva Pharmaceutical Industries (Petah Tikva, Israel) because of the development of potentially inactivating antibodies. Somavaratan developed by Versartis (Menlo Park, USA) was a growth hormone molecule fused with hydrophilic strings of amino acids that prolonged serum half-life. It was discontinued when a phase 3 study revealed it to be inferior to daily recombinant human growth hormone ( 41 ).

## CONCLUSIONS

The pharmaceutical industry has spent many years and hundreds of millions of dollars betting that a long-acting growth hormone preparation can be devised that is non-inferior to daily growth hormone and that it will improve adherence to treatment. A summary of long-acting growth hormone preparations is presented in Table 1 . While many of these preparations have not been shown to be effective or practical ( 42 ), two drugs are currently being marketed in Asia and several more are on the verge of being considered for regulatory approval by agencies in the United States and Europe. A number of important questions have yet to be answered. While we know that long-term recombinant human growth hormone therapy is very safe ( 43 ), it is not known whether the various long-acting preparations will be equally safe. Will years of continuous non-pulsatile growth hormone exposure lead to acromegalic changes? Will the moieties added to fusion proteins lead to a new set of side effects that are currently unimagined? It will be incumbent upon each company to perform extensive post-marketing surveillance to determine the safety of each of these novel preparations ( 44 ). And since the original rationale for developing a long-acting preparation was improved drug adherence, the companies should study if their growth hormone preparations do indeed lead to better long-term results.

**Table 1:** Summary of long-acting recombinant human growth hormone preparations

Class	Company	Product	Design	Status
**Depot Formulations**				
	Genentech (South San Francisco, USA) Alkermes (Dublin, Ireland)	Nutropin Depot	Encapsulated in Polylactide-Coglycolide Microspheres	FDA approved, but removed from the market in 2004.
	BioPartners (Los Angeles, USA) LG Life Sciences (Seoul, South Korea)	Declage LB03002	Growth hormone incorporated into sodum hyaluronate and suspended in MCT for injection	Approved for pediatric growth hormone deficiency in South Korea
**PEGylated Formulations**				
	Ambrx (La Jolla, USA)	ARX201	30-kDa PEG incorporated into recombinant human growth hormone	Drug development stopped due to PEG accumulation in ependymal cells of choroid plexus
	Novo Nordisk (Bagsvaerd, Denmark)	NNCI126-0083	43-kDa PEG linked to recombinant human growth hormone	Drug development stopped
	Pfizer (New York City, USA)	PHA-794428	Branched 40-kDa PEG linked to amino end of recombinant human growth hormone	Drug development stopped
	GeneScience Pharmaceuticals (Changchun, China)	Jintrolong	40-kDa PEG linked to growth hormone	Approved for pediatric growth hormone deficiency in China
**Pro-drug Formulations**				
	Ascendis Pharma (Hellerup, Denmark)	TransCon Growth Hormone	Recombinant human growth hormone transiently bound to mPEG	Phase 3 testing concluding. Seeking regulatory approval in 2020
**Non-Covalent Albumin Binding Growth Hormone**				
	Novo Nordisk (Bagsvaerd, Denmark)	Somapacitan	Recombinant human growth hormone with a single point mutation attached to a terminal fatty acid that reversibly binds albumin	Ongoing pediatric phase 3 testing
**Growth Hormone Fusion Proteins**				
	Genexine (Seongnam, South Korea) Handok (Seoul, South Korea)	HyTropin GX-H9	Recombinant human growth hormone fused to hybrid Fc	Pending phase 3 trial
	OPKO Health (Miami, USA) Pfizer (New York City, USA)	Somatrogon Lagova MOD-4023	Recombinant human growth hormone fused to 3 copies of carboxy-terminal of hCG’s beta subunit	Phase 3 testing in children underway.
	Teva (Petah Tikva, Israel)	TV-1106	Recombinant human growth hormone fused to albumin	Blocking/inactivating antibodies developed, drug abandoned.
	Versartis (Menlo Park, USA)	Somavaratan VRS-317	Recombinant human growth hormone fused to hydrophilic strings of amino acids	Discontinued due to inferiority to daily recombinant human growth hormone in increasing adult height
